# Perceived quality of teaching and course engagement in entrepreneurship education: the moderating role of teacher interpersonal behaviour among first-year undergraduates at a Chinese university

**DOI:** 10.3389/fpsyg.2026.1936338

**Published:** 2026-10-19

**Authors:** Dake Lin

**Affiliations:** 1 Public Administration Office, Wenzhou University, Wenzhou, Zhejiang, China; 2 Faculty of Education, Languages, Psychology and Music, SEGi University, Petaling Jaya, Selangor, Malaysia

**Keywords:** entrepreneurship education, first-year undergraduates’ students, perceived quality of teaching, student course engagement, teacher interpersonal behaviour

## Abstract

This cross-sectional, single-institutional study investigates associations between perceived quality of teaching (PQT) and first-year undergraduates’ student course engagement (SCEn) within entrepreneurship education and examines the statistical moderating role of teacher interpersonal behaviour (IB). Survey data of 456 first-year undergraduates from 16 schools at one provincial university were analysed via Partial Least-Squares Structural Equation Modelling (PLS-SEM). Results show a substantial positive cross-sectional association between PQT and SCEn (*β* = 0.516, *p* < 0.001). IB also exhibits a positive statistical association with SCEn (*β* = 0.403, *p* < 0.001). Teacher IB significantly moderates the PQT-SCEn association, yet the magnitude of this moderating effect is modest (*β* = 0.068, *p* = 0.038). These correlational findings suggest that student course engagement is correlated with both perceived quality of teaching and teacher interpersonal behaviour. For entrepreneurship-education practice, the findings point to the value of integrating pedagogical improvement with supportive interpersonal teaching practices to foster student course engagement and inform curriculum design and faculty development.

## Introduction

Entrepreneurship education (EE) has gained increasing global recognition as a strategic instrument for promoting economic growth, innovation, and employment generation (Fan et al., 2022). EE has a long scholarly and practical tradition; its earliest university courses emerged in the mid-twentieth century, decades prior to international policy formalization in the late 1980s (Katz, 2003). Since then, it has been framed as a core element of national development strategies alongside vocational and academic education (Suguna et al., 2024). Over the past decades, higher education institutions worldwide have systematically incorporated EE into their curricula to enhance students’ entrepreneurial competencies, employability, and adaptability in uncertain labor markets (Gazi et al., 2024).

Around the world, higher-education entrepreneurship education targets core entrepreneurial competencies including self-efficacy, creativity, risk-taking and innovativeness, supporting global sustainable development agendas (Branca et al., 2025). Building on this global evidence base, this study further focuses on the Chinese higher-education setting. In China, the rapid expansion of EE has been strongly driven by national innovation policies and increasing entrepreneurial activities (Mei and Symaco, 2022). Statistics from the National Bureau of Statistics of China (2026), 25.74 million new market entities were established in 2025, with a daily average of 26,000 newly registered enterprises, reflecting entrepreneurship’s growing importance as a viable career pathway. Despite this strategic emphasis, persistent concerns remain regarding the effectiveness of EE teaching and students’ engagement in entrepreneurship courses.

Previous studies indicate relatively low student course engagement in compulsory entrepreneurship education in Chinese universities (Knox, 2022; Wang et al., 2021). Empirical evidence suggests that many students perceive entrepreneurship courses as less academically rigorous or less relevant than traditional disciplinary subjects, which leads to inattentiveness, surface learning, and limited classroom participation. Some students even regard entrepreneurships as a “last resort” career option rather than a proactive professional choice, further weakening their motivation to engage meaningfully in EE classrooms. Such disengagement not only undermines learning outcomes but also challenges the broader educational objective of cultivating entrepreneurial mindsets and competencies among university students. Consequently, scholars increasingly emphasize that improving instructional quality and classroom learning environments is essential for addressing engagement-related problems in EE.

Within this context, perceived quality of teaching (PQT) has been widely acknowledged as a central determinant of students’ course engagement (SCEn). High-quality teaching—characterized by clear learning objectives, appropriate workload, effective assessment, and interactive pedagogical strategies—can foster students’ cognitive, emotional, and behavioral engagement by creating a stimulating learning environment. Beyond instructional design, teacher–student interpersonal behaviour (IB) has also been identified as a critical motivational factor influencing students’ classroom participation, learning persistence, and overall engagement. Prior studies highlight that student-centered teaching approaches and supportive interpersonal interactions enhance students’ learning experiences and course satisfaction in EE (Zhang and Mei, 2020). Moreover, a growing body of research indicates that positive classroom climates and constructive teacher–student relationships can strengthen students’ engagement and entrepreneurial intentions (Gao and Qin, 2022; Sim et al., 2023; Zhou, 2021).

Despite these insights, several important research gaps remain insufficiently addressed. First, although a positive association between teaching quality and student engagement has been well documented, the specific relationship between PQT and SCEn within entrepreneurship education remains theoretically and empirically underdeveloped (An et al., 2022). In particular, it is unclear whether higher perceived quality of teaching directly translates into stronger engagement among EE students, especially at the early undergraduate stage. Second, while recent studies suggest that teachers’ interpersonal behaviour may moderate the relationship between teaching quality and student engagement (Tian and Shen, 2023; Zhang, 2022), existing evidence is largely derived from contexts such as physical education or language learning. Whether this moderating mechanism operates similarly in entrepreneurship education has not yet been systematically examined, leaving a clear contextual gap in the literature.

Third, prior EE research has tended to focus on curriculum design, entrepreneurial intention, or policy-level outcomes, while largely overlooking classroom-level psychological and relational mechanisms that shape students’ learning engagement. For instance, Fan et al. (2022) demonstrated that student course engagement accounts for nearly 34% of the total effect on perceived EE quality; however, they did not explicate how PQT contributes to engagement, nor did they consider the potential moderating role of teacher interpersonal behaviour. Similarly, studies on compulsory courses indicate that unengaging content, limited interaction, and one-way communication reduce students’ attention and participation (Wang, 2022), yet these studies did not situate their analyses within entrepreneurship education or explore the interactive effects of PQT and IB.

Additionally, long-standing problems related to EE teaching quality further reinforce the need for focused investigation. Research has identified issues such as weak course organization, insufficient pedagogical innovation, limited responsiveness to student feedback, and ineffective teacher–student interaction in EE classrooms (Tang and Zhang, 2018). These problems have contributed to declining student perceptions of teaching quality and low willingness to recommend EE courses, ultimately leading to reduced academic course engagement (Ma and Wang, 2021). In the Chinese context, these challenges are compounded by the widespread practice of assigning out-of-field teachers to teach entrepreneurship courses. Although EE requires a balance of theoretical knowledge and practical experience, many instructors lack relevant entrepreneurial backgrounds or training, which constrains both instructional quality and interpersonal effectiveness (Fan et al., 2022).

This study focuses on the Chinese higher education context. In China, entrepreneurship education is universally compulsory for undergraduate students, forming a unique, standardized educational environment. Meanwhile, Chinese classrooms feature prominent relational cultural characteristics, where teacher–student interpersonal interactions profoundly shape student learning attitudes and behavioral engagement. This context provides a valuable and representative setting to explore how classroom factors jointly influence student engagement and entrepreneurial development.

Taken together, existing studies have not fully clarified how perceived quality of teaching and teacher interpersonal behaviour jointly influence student course engagement in entrepreneurship education, particularly among first-year undergraduates in China. This unresolved issue represents a critical gap, given the contemporary expansion of EE and its strategic role in innovation-driven development. Addressing this gap is essential not only for advancing EE theory but also for improving instructional practices and learning outcomes in real educational settings.

Accordingly, this study seeks to investigate the relationship between perceived quality of entrepreneurship course teaching and student course engagement, while further examining the moderating effect of teacher interpersonal behaviour. By focusing on first-year undergraduates from 16 schools in China, this research responds directly to contemporary challenges in EE implementation and contributes new empirical evidence to the literature by integrating instructional quality and interpersonal dynamics within a unified analytical framework.

Specifically, this study addresses the following research questions:

1. To what extent is perceived quality of entrepreneurship course teaching associated with student course engagement among first-year undergraduates?
2. To what extent does teacher interpersonal behaviour moderate the relationship between perceived quality of entrepreneurship course teaching and student course engagement among first-year undergraduates?

## Literature review and theoretical framework

Entrepreneurship education (EE) occupies a core position within contemporary higher-education policy and curriculum design, as it aims to nurture not only venture-creation skills but also generic entrepreneurial competencies including innovation, adaptive problem-solving, and employability for broad student populations (Nguyen and Nguyen, 2023; Shen et al., 2025).

### Perceived quality of teaching in EE

Entrepreneurship education has increasingly shifted from traditional knowledge transmission toward experiential and practice-oriented pedagogies. Contemporary research suggests that experiential learning better aligns with the competence-based goals of entrepreneurship programs, whereas purely lecture-based approaches are gradually perceived as insufficient (Eisenstein et al., 2021). Empirical evidence indicates that entrepreneurship education effectiveness is reflected in enhanced entrepreneurial competencies, reduced perceived barriers, and strengthened entrepreneurial intentions (Liu et al., 2021).

In the Chinese context, structural and cultural factors complicate this transformation. Although entrepreneurship education has been formally integrated into undergraduate evaluation systems (The Ministry of Education of China, 2012), instructional quality remains uneven. A large proportion of instructors are part-time or lack systematic training (Wang, 2023), and rapid policy expansion has outpaced faculty development (Gao and Qin, 2022). These conditions may weaken students’ perceptions of teaching quality, particularly when theoretical instruction is insufficiently connected to real entrepreneurial practice.

Perceived Quality of Teaching (PQT) in entrepreneurship courses typically encompasses instructional content, teaching methods, management, and learning conditions (Gao et al., 2021). However, despite extensive discussion of course reform and policy development, limited research has empirically linked students’ perceived quality of teaching with their academic course engagement. Addressing this gap is particularly important in entrepreneurship education, where engagement is essential for translating pedagogical design into competence development.

### Student course engagement in EE

Student course engagement (SCEn) is a critical prerequisite for meaningful entrepreneurship-education outcomes, as entrepreneurial competencies cannot be passively acquired via lecture-based teaching (Knox, 2022; Liu et al., 2023). In compulsory EE, high multi-faceted engagement facilitates experiential learning and entrepreneurial psychological development, whereas low engagement may render mandatory courses symbolic. Empirical evidence identifies course engagement as a key intermediate mechanism translating instructional inputs into desirable EE results (Liu et al., 2023). SCEn reflects students’ behavioral, emotional, and cognitive investment in academic learning. Research consistently demonstrates that interactive instructional strategies and active learning environments enhance engagement and academic outcomes (Cole et al., 2021; Freeman et al., 2020). In entrepreneurship education, experiential projects and real-world simulations further strengthen perceived relevance and motivation (Nabi et al., 2017).

Nevertheless, engagement in entrepreneurship courses cannot be explained solely by pedagogical design. Students’ perceptions of teaching quality and the interpersonal classroom climate shape whether instructional inputs are internalized as motivational resources. This suggests that engagement is not merely an outcome of course structure but is mediated by students’ interpretations of instructional and relational quality.

### Teacher interpersonal behaviour in EE

Teacher interpersonal behaviour represents the relational climate within which teaching quality is enacted. Empirical research demonstrates that supportive, leadership-oriented, and proximity-based teacher behaviours are associated with stronger emotional and cognitive engagement across disciplines (van Uden et al., 2014). Conversely, even subtle controlling behaviours may frustrate students’ psychological needs and undermine engagement (Tilga et al., 2020).

In Chinese entrepreneurship education, hierarchical traditions and directive pedagogies may conflict with the autonomy-supportive orientation required for entrepreneurial learning (Mei and Symaco, 2022). Given rapid program expansion and uneven teacher preparation (Gao and Qin, 2022), interpersonal behaviour may function as a crucial boundary condition that strengthens or weakens the translation of perceived quality of teaching into student engagement. Yet empirical evidence on this moderating role in entrepreneurship courses remains scarce.

### Theoretical framework

Although three theoretical perspectives inform this study, Self-Determination Theory (SDT) serves as the primary theoretical foundation, given its direct alignment with our measured teacher interpersonal behaviour construct. Self-Efficacy Theory and Incremental Theory of Learning are retained merely as complementary background perspectives. Importantly, these two corresponding constructs are not measured in the present study; accordingly, our dataset cannot empirically test their underlying psychological mechanisms.

*Self-Determination Theory (SDT)* (Ryan and Deci, 2000) offers the primary motivational lens. SDT posits that engagement flourishes when classroom environments satisfy students’ needs for autonomy, competence, and relatedness. High perceived quality of teaching may support competence development, but its motivational impact depends on whether interpersonal behaviour is perceived as autonomy-supportive rather than controlling. The Interpersonal Behaviour Questionnaire (Rocchi et al., 2017), grounded in SDT, operationalizes these relational dynamics. From this perspective, teacher interpersonal behaviour moderates the PQT–SCEn relationship by shaping the extent to which instructional quality activates intrinsic motivation.

*Self-Efficacy Theory (SET)* (Bandura, 1997) further explains the psychological mechanism linking instructional and relational factors to engagement. Positive teacher–student relationships enhance students’ confidence in their learning capabilities, thereby strengthening engagement (Hajovsky et al., 2020; Yang and Li, 2021). High-quality instruction combined with supportive interpersonal behaviour may provide mastery experiences and constructive feedback that reinforce academic self-efficacy.

*Incremental Theory of Learning (ITL)* (Dweck, 1999) serves as a foundational assumption underlying engagement tendency. Students who believe abilities can be developed through effort exhibit higher levels of emotional and cognitive engagement (Handelsman et al., 2005). In this study, ITL supports the conceptualization and measurement of student course engagement rather than introducing an additional causal pathway.

Taken together, these three theories are not merely general educational perspectives imported from outside the entrepreneurship field. SDT, SET and ITL collectively illuminate the intertwined relationships among perceived teaching quality, teacher interpersonal behaviour (IB) and student course engagement (SCEn), specifically within the compulsory entrepreneurship-education (EE) setting of Chinese universities. SDT accounts for the motivational moderating function of IB; SET highlights confidence-building pathways toward SCEn; ITL explains students’ willingness to engage in EE activities under favourable teaching conditions. This integrated theoretical lens informs the hypotheses developed in the subsequent section.

## Hypothesis development

### Perceived quality of teaching and student course engagement

Perceived Quality of Teaching (PQT) captures students’ evaluations of instructional clarity, organization, responsiveness, and pedagogical effectiveness (Rollett et al., 2021). In higher education, students’ perceptions of teaching quality consistently predict academic engagement (Qureshi et al., 2023). When instruction is perceived as well-structured and intellectually stimulating, students are more likely to invest cognitively and emotionally in coursework.

This relationship may be particularly salient in entrepreneurship education, where experiential and autonomy-oriented learning requires sustained student involvement. Active learning and real-world problem solving have been shown to enhance engagement in such contexts (Mei et al., 2020). Despite this theoretical plausibility, empirical evidence examining the direct association between PQT and student course engagement (SCEn) in entrepreneurship education remains limited.

Perceived quality of teaching (PQT) captures students’ evaluations of instructional clarity, organization, responsiveness, and pedagogical effectiveness (Rollett et al., 2021). From the perspective of Self-Determination Theory and Incremental Theory of Learning, well-structured high-quality teaching satisfies students’ competence needs and reinforces the belief that entrepreneurial-related abilities can be improved through learning (Dweck, 1999; Ryan and Deci, 2000). Prior higher-education empirical studies consistently report positive links between perceived teaching quality and different forms of student engagement (Qureshi et al., 2023). Nevertheless, such empirical evidence is scarce within compulsory entrepreneurship education, where learning relies heavily on experiential practice rather than traditional disciplinary instruction. Given this theoretical reasoning and indirect empirical support, we expect higher perceived teaching quality to predict greater student course engagement.

*H1*: Perceived quality of entrepreneurship course teaching is positively associated with student course engagement.

### The moderating role of teacher interpersonal behaviour

Teacher interpersonal behaviour (IB) shapes the relational climate in which instructional quality is interpreted by students. According to Self-Determination Theory, the motivational benefits of high-quality teaching are not automatic; they depend on whether the interpersonal context provides autonomy and relatedness support (Ryan and Deci, 2000). Self-Efficacy Theory further suggests that supportive teacher-student interactions can strengthen learners’ confidence, amplifying how students draw value from instructional inputs (Bandura, 1997). Empirically, comparable moderating effects of teacher interpersonal dimensions have been documented in physical education and language-learning contexts (Liu, 2021; Tian and Shen, 2023). However, this conditional mechanism remains under-examined within entrepreneurship education, where uncertainty and creative tasks make students particularly sensitive to interpersonal support. Therefore, we propose that teacher interpersonal behaviour will strengthen the positive association between PQT and SCEn.

*H2*: Teacher interpersonal behaviour moderates the relationship between perceived quality of entrepreneurship course teaching and student course engagement.

## Methodology

### Research procedure and samples

The study sample comprised first-year undergraduates from 16 schools at a provincial university in China with a strong institutional emphasis on entrepreneurship education. The university enrolls students from diverse disciplinary and regional backgrounds, providing an appropriate context for examining perceived quality of teaching, teacher interpersonal behaviour, and student course engagement.

Ethical approval was obtained from the SEGi Research Ethics Committee (Approval No.: SEGiEC/SR/FOELPM/7/2024–2025, approval date: 2 July 2024) prior to data collection. An information letter outlining the research purpose and procedures was sent to the heads of Student Affairs in each school. Upon approval, data collection sessions were scheduled either face-to-face or online. Participants were informed of the study objectives before completing the survey.

A total of 456 students completed the questionnaire via Questionnaire Star distributed through WeChat groups. The instrument consisted of four sequential sections.

The unit of analysis in this study is first-year students at a comprehensive university. The criteria for determining individuals eligible to participate in the study were as follows: (1) students must be in their first year of study; (2) they must have completed the mandatory undergraduate entrepreneurship course; (3) they must be enrolled in one of the 16 schools; and (4) their nationality must be Chinese. The study employed a combination of stratified and convenience sampling techniques, and data were collected cross-sectionally through an online survey using Questionnaire Star. Stratified sampling was first applied based on academic disciplines to ensure adequate representation of students from Humanities and Social Sciences, Engineering and Technological Sciences, and Natural Sciences. Within each stratum, convenience sampling was employed due to administrative constraints and the large cohort size of first-year students. This combined sampling strategy was considered appropriate given the exploratory nature of the study and the practical constraints of accessing a large first-year cohort. While convenience sampling may limit strict randomization, the initial stratification and full coverage of all schools helped mitigate potential sampling bias.

Each student from the 16 schools received a Questionnaire Star link containing a questionnaire, which was accompanied by a cover letter outlining the study’s purpose, confidentiality assurances, and the voluntary nature of participation. Between January and May 2024, a total of 456 first-year students from 16 schools were invited to participate. All invited students voluntarily completed the questionnaire after informed consent, resulting in a 100% response rate.

All 456 eligible first-year undergraduates across 16 schools were approached for this study. Participation was entirely voluntary. Prior to survey distribution, participants received an information sheet explaining study objectives, anonymity, and their right to decline participation without any negative consequences. Consent was implied upon opening the online questionnaire. No faculty members were present while students completed the web-based survey. Of the 456 eligible participants, 456 consented and completed the questionnaire; zero responses were excluded due to incomplete or careless responding. The final analytical sample contained *N* = 456. Missing values were handled using the default PLS-SEM treatment for partial item non-response. Quality control included visual screening for patterned straight-lining response behaviour. Official university channels were only used to forward the survey invitation link, and faculty supervision referred solely to logistical coordination rather than pressuring individuals to take part. The sample size of 456 meets the requirements for reliable PLS-SEM analysis (Hoogland and Boomsma, 1998; Kline, 2016).

An analysis of respondents’ demographic information revealed that among the 456 participants, 45.8% were male, while 54.2% were female. The majority of respondents were 19 years old (51.8%), followed by 18 years old (25.2%) and 20 years old (23.0%). In terms of academic disciplines, 47.4% of students were from Humanities and Social Sciences, 30.9% from Engineering and Technical Sciences, and 21.7% from Natural Sciences.

Although the sample was drawn from a single comprehensive university, several features enhance its representativeness. First, the university covers all three major disciplinary categories defined by the national academic classification system, ensuring disciplinary diversity. Second, students were recruited from 16 schools and originate from over 30 provinces and municipalities, reflecting broad regional and socio-demographic variation. Therefore, findings from this sample are generalizable primarily to first-year undergraduates taking compulsory entrepreneurship courses within this sampled university. Although participants originate from multiple provinces, individual-level geographic diversity does not guarantee representativeness for comprehensive universities across China, though the research framework and analytical approach may be extended to similar types of higher education institutions for reference.

Nevertheless, caution should be exercised when generalizing the findings to other institutional contexts, such as vocational colleges or specialized universities.

### Research instruments

To evaluate the impact of Perceived Quality of Teaching on Student Course Engagement and examine the moderating role of Teacher Interpersonal Behaviour, this study employed a quantitative cross-sectional survey. The questionnaire consisted of two sections: the first collected participants’ demographic characteristics, while the second included three measurement scales, as follows.

#### Perceived quality of teaching

The study adapted the Student Course Experience Questionnaire (SCExQ) made by Ginns et al. (2007) for the context of compulsory entrepreneurship courses and renamed it the Perceived Quality of Teaching Questionnaire (PQTQ). Note that the original SCExQ dimensions extend beyond narrowly-defined teaching quality and some original items refer to the whole degree programme. We therefore systematically revised item stems to target the specific entrepreneurship course rather than the broader degree programme.

This constitutes substantial contextual adaptation; accordingly, PQTQ is treated as an adapted scale in the present study instead of a direct adoption of the original SCExQ. Full retained item wording (Chinese and back-translated English), item-level modification log, and marked reverse-scored items are documented in Supplementary Tables S1, S2. Content validity was examined via CVR assessment, and cross-cultural equivalence was established through translation-back-translation procedures described below.

Full item wording, item-level modification log are presented in Supplementary Tables S1, S2.

#### Student course engagement

We used the Student Course Engagement Questionnaire (SCEnQ) from Handelsman et al. (2005) to assess student course engagement. The original scale includes four dimensions (Skills, Emotional, Participation/Interaction, Performance) with 23 items. This scale fits well with the participatory and practical features of entrepreneurship courses. Responses were recorded on a five-point Likert scale (1 = “Strongly Disagree,” 5 = “Strongly Agree”).

Full wording of retained items can be found in Supplementary Table S3.

#### Teacher interpersonal behaviour

The study adopted the Interpersonal Behaviour Questionnaire developed by Rocchi et al. (2017) to measure teacher interpersonal behaviour, which have 24 items. The instrument consists of six dimensions, namely Autonomy support (4 items), Autonomy thwarting (4 items), Competence support (4 items), Competence thwarting (4 items), Relatedness support (4 items), and Relatedness thwarting (4 items). Items were rated on a five-point Likert-type scale (1 = “Strongly Disagree,” 5 = “Strongly Agree”).

Retained items and marking for reverse-scored items are available in Supplementary Table S4.

In this study, items measuring need-thwarting behaviours were reverse-coded before aggregation, so higher total IB scores represent overall higher levels of perceived adaptive interpersonal behaviours. Although the original IBQ contains six distinct sub-dimensions, we treat the retained items collectively as a first-order aggregate-level latent construct in the subsequent PLS-SEM measurement model, instead of specifying a higher-order factor structure. The reliability and convergent validity results for this aggregated latent construct are presented in Table 1.

**Table 1:** Convergent validity results of PQT, SCEn, and IB.

Variable	Items	FL	rho_A	CR	AVE	Alpha (*α*)
Perceived Quality of Teaching (PQT)	PQT1	0.803	0.960	0.959	0.583	0.959
	PQT2	0.821				
	PQT3	0.793				
	PQT4	0.781				
	PQT6	0.769				
	PQT7	0.785				
	PQT8	0.794				
	PQT9	0.686				
	PQT15	0.403				
	PQT16	0.437				
	PQT18	0.796				
	PQT19	0.812				
	PQT20	0.833				
	PQT21	0.853				
	PQT22	0.82				
	PQT23	0.844				
Student Course Engagement (SCEn)	SCEn1	0.708	0.943	0.942	0.536	0.936
	SCEn2	0.76				
	SCEn3	0.598				
	SCEn4	0.59				
	SCEn9	0.504				
	SCEn10	0.811				
	SCEn11	0.802				
	SCEn12	0.793				
	SCEn14	0.792				
	SCEn15	0.683				
	SCEn17	0.798				
	SCEn19	0.724				
	SCEn20	0.771				
	SCEn21	0.737				
	SCEn23	0.823				
Teacher Interpersonal Behaviour (IB)	IB1	0.802	0.953	0.945	0.518	0.955
	IB2	0.853				
	IB3	0.867				
	IB4	0.84				
	IB7	0.445				
	IB8	0.443				
	IB9	0.72				
	IB10	0.835				
	IB11	0.82				
	IB12	0.803				
	IB17	0.733				
	IB18	0.79				
	IB19	0.799				
	IB20	0.819				
	IB21	0.412				
	IB23	0.437				
	IB24	0.458				

FL, standardized factor loading; rho_A is the consistent reliability estimator for PLS-SEM; CR, composite reliability; AVE, average variance extracted.

Prior to the main study, a pilot test was conducted to assess reliability and validity. Five experts in the field of academic entrepreneurship participated in the content validity assessment to determine whether the measurement items accurately captured the intended constructs. A panel of 3–5 experts is commonly reported for CVR-based content validity assessment in educational survey adaptation studies (Polit and Beck, 2006), The expert panel was contacted via email or postal mail with printed materials, and the research team refined certain measurement items based on their feedback. This ensured clarity and comprehensibility within the research context, thereby enhancing the overall integrity of the questionnaire.

*A priori* cut-off for item retention was set prior to main-data collection. Given a panel of five experts, items with CVR > 0 were tentatively retained for further assessment; items with negative CVR were eliminated immediately. We acknowledge that CVR = +0.2 represents marginal content validity, so these marginally-retained items were subjected to subsequent PLS-SEM measurement-model screening based on outer loading threshold (0.4), rather than being accepted solely on positive CVR.

The questionnaire was initially translated into Chinese. Recognizing the equivalence issues in instrument translation between cultures (Davidov et al., 2014), the translation of instruments in this study followed the procedures recommended by Zamanian et al. (2024). The questionnaire was first translated into Chinese by language experts proficient in both English and Chinese. The translated questionnaire was then back translated into English by another proficient language teacher. A third language teacher cross-checked the back-translated questionnaire with the original one. Finally, both the English and Chinese versions of the questionnaire were thoroughly reviewed by two expert team members proficient in both languages.

Items eliminated based on negative CVR scores and subsequent PLS-SEM-based item screening are fully documented in Supplementary Tables S1–S4.

The questionnaire items were evaluated by a panel of five experts, each of whom was asked to assess every item. The rating scale consisted of two options: “essential and useful” or “nonessential and unnecessary.” To determine whether an item should be retained, revised, or removed, the content validity ratio (CVR) was calculated using the following formula:

$$\mathrm{CVR}=(n_{e}-N/2)/(N/2)$$

n_e_ = number of panelists indicating “essential,” N = total number of panelists.

The CVR ranged from −1 to +1, with positive values indicating that more than half of the panelists rated an item as “essential.” In this study, the CVR was positive for all questionnaire items except for thirteen (Questions 5, 10, 13, and 17 in PQTQ; Questions 5–8, 13, 16, 18 and 22 in SCEnQ, Questions 22 in IBQ), which were excluded (see Table 2). The CVR test results, summarized in Table 2, show that the retained items had CVR values ranging from +0.2 to +1.

**Table 2:** First-round CVR calculations for questionnaire items.

Questions	n_e_	CVR	Interpretation
PQTQ 1, 3–4, 7, 12, 14, 19–21	5	+1	Remained
PQTQ 2, 6, 8–9, 11, 18, 22–23	4	+0.6	Remained
PQTQ 15, 16	3	+0.2	Remained
PQTQ 5, 10, 13	2	−0.2	Eliminated
PQTQ 17	1	−0.6	Eliminated
SCEnQ 2, 9–11, 14, 17, 19, 21, 23	5	+1	Remained
SCEnQ 3, 12, 15, 20	4	+0.6	Remained
SCEnQ 4, 1	3	+0.2	Remained
SCEnQ 5–8, 22	2	−0.2	Eliminated
SCEnQ 13, 16, 18	1	−0.6	Eliminated
IBQ 1–2, 4, 6, 8–12, 14, 16–20, 23–24	5	+1	Remained
IBQ 3, 5, 13, 15, 21	4	+0.6	Remained
IBQ 7	3	+0.2	Remained
IBQ 22	2	−0.2	Eliminated

CVR, Content Validity Ratio. This table only summarizes expert CVR evaluation outcomes and does not represent the final item set for the PLS-SEM model. Items with negative CVR values were excluded before main-sample data collection. A further subset of items with positive CVR values were subsequently removed during PLS-SEM measurement-model assessment due to outer-loading values below the 0.4 threshold. Full exhaustive item-level tracking (original identifiers, adapted wording, CVR results, pre-deletion loadings, removal rationale, reverse-coding status, and final-model inclusion status) is reported in Supplementary Tables S1–S4.

Face validity refers to the extent to which a measurement instrument appears to assess the intended content. It is typically evaluated through intuitive judgment or participant feedback (DeVon et al., 2007). This study conducted a face validity assessment with five experts in entrepreneurship education and five first-year students to gather feedback on the questionnaire. The results indicated that all participants comprehended the survey items.

Finally, to further assess the validity of the study, the research team conducted a pilot test by distributing the questionnaire to a sample of 127 first-year students. The results indicated that the Cronbach’s α coefficients for all measured constructs exceeded 0.70 (Hair et al., 2014). Specifically, the reliability coefficients were as follows: Perceived Quality of Teaching (*α* = 0.939), Student Course Engagement (*α* = 0.993), and Teacher Interpersonal Behaviour (*α* = 0.916). After validation, all measurement items were deemed suitable for further formal analysis.

The pilot-test Cronbach’s *α* of 0.993 for student course engagement is notably high. Such near-unity reliability may signal potential item redundancy or patterned participant responding, rather than merely excellent internal consistency. We reviewed item content and observed partial wording overlap across items, though each item taps distinct sub-dimensions of behavioural, emotional and cognitive engagement. This potential limitation is acknowledged in the present study.

### Data analysis

We employed Partial Least Squares-Structural Equation Modelling (PLS-SEM) for several methodological reasons rather than relying merely on sample size. First, this study emphasizes prediction-oriented analysis to examine the predictive effect of teaching quality and teacher interpersonal behaviour on student course engagement, which fits the strength of PLS-SEM. Second, several reflective indicators have outer loadings in the marginal 0.4–0.7 range after substantial scale adaptation; PLS-SEM is more robust for models containing indicators with marginal psychometric performance. Third, our model includes a latent interaction term (PQT × IB) for testing moderation, and PLS-SEM is well-suited for estimating latent variable interaction effects. Covariance-based SEM (CB-SEM) was not prioritized because it focuses on theory confirmation rather than prediction and is less tolerant of marginally-performing adapted-scale indicators. SmartPLS 4.0 was used for all estimations. Bootstrap was run with 5,000 BCa resamples at the 95% confidence level.

#### Testing the assumptions of multivariate analysis

The collinearity statistics (Table 3) indicate that the tolerance values for all predictors are above 0.1 (Norušis, 1982), while the VIF values are below 10 (Kutner et al., 2005). Therefore, we conclude that there is no evidence of multicollinearity among the predictor variables in this study, indicating that the independent variables in our research model are not highly correlated.

**Table 3:** Collinearity statistics (VIF) for predictors of student course engagement (SCEn).

Model	Collinearity statistics	
	Tol	VIF
Perceived quality of teaching (PQT)	0.368	2.715
Teacher interpersonal behaviour (IB)	0.380	2.632
PQT × IB (interaction term)	0.881	1.135

Tol, Tolerance; VIF, Variance Inflation Factor. VIF values below 5 indicate no critical multicollinearity issue.

#### The profiles of the respondents of this study

This study was conducted in 16 different schools and three different disciplines (Table 4) in a provincial university in the Zhejiang Province, China. According to the National Standard Discipline Classification and Code of the People’s Republic of China.

**Table 4:** Demographic profiles of the respondents of this study (*N* = 456).

Demographic	Characteristics	Number of students
Gender	Male	209(45.8%)
	Female	247(54.2%)
Age	18	115(25.2%)
	19	236(51.8%)
	20	105(23.0%)
Engineering and Technological Science	School of Electrical & Electronic Engineering	24
	School of Mechanical & Electrical Engineering	27
	School of Computer & Artificial Intelligence	30
	School of Architectural Engineering	22
Humanities and Social Sciences	School of Business	26
	School of Humanities	21
	School of Foreign Languages	24
	School of Music	24
	School of Physical Education & Health	21
	School of Marxism	14
	School of Education	60
	School of Fine Arts & Design	26
	School of Law	38
Natural Science	School of Mathematics & Physics	24
	School of Chemistry & Materials	51
	School of Life & Environmental Science	24
	Total	456

Based on on stratified sampling on gender and discipline, a total of 103 participants (22.6%) were selected from the Engineering and Technical Sciences, 254 (55.7%) from the Humanities and Social Sciences, and 99 (21.7%) from the Natural Sciences. Overall, the number of participants for each school ranged from 14 to 60 with School of Marxism having 14 participants and School of Education having 60.

#### Descriptive statistics of the research variables

Descriptive statistical analysis was employed to determine the mean and standard deviation for each survey item within the primary constructs (see Table 5). The overall mean score for Perceived Quality of Teaching (PQT) was *M* = 3.97, SD = 0.61 (Sekaran, 2010). Overall, the students’ perceived quality of teaching was close to 4.0, indicating that most students held positive attitudes toward the teaching content, instructional methods, and learning support. While the overall perception of teaching quality was favorable, attention should be given to groups with lower scores. By refining instructional approaches and course design, further enhancements can be made to optimize students’ learning experiences and ensure the consistency of teaching quality.

**Table 5:** The descriptive analysis score for PQT, SCEn, and IB.

Variable	Mean	Standard deviation (SD)	Score
Perceived quality of teaching (PQT)	3.97	0.61	High
Student course engagement (SCEn)	3.94	0.63	High
Teacher interpersonal behaviour (IB)	4.02	0.48	High

Overall, the students’ level of student course engagement was close to 4.0, as indicated by a mean score of *M* = 3.94, SD = 0.63 (Sekaran, 2010). This suggests that most students are actively engaged in their academic activities and demonstrate positive attitudes toward course content, teaching practices, and institutional support. Although the overall engagement level is promising, special attention should be paid to subgroups with lower scores. By implementing targeted engagement strategies and refining course design, further improvements can be achieved to enhance students’ overall academic involvement and ensure a more consistent learning experience.

The overall mean score for Teacher Interpersonal Behaviour (IB) was *M* = 4.02, SD = 0.48 (Sekaran, 2010). Overall, the students’ perceived level of teacher interpersonal behaviour was above 4.0, indicating that most students experienced highly effective and supportive interactions from their teachers. While the overall perception of teacher interpersonal behaviour was very positive, attention should still be given to ensuring consistency across all student groups. By enhancing teacher communication strategies and fostering a more engaging classroom environment, further improvements can be made to optimize the quality of teacher-student interactions and enrich the overall learning experience.

#### Measurement model

Given substantial contextual adaptation of the PQTQ scale, the convergent and discriminant validity reported in Tables 1, 6 serve as renewed construct-validation evidence for this adapted instrument. All three multi-dimensional scales in this study are operationalized as first-order aggregate-level latent constructs, with their psychometric properties reported in Table 1. Partial least squares structural equation modeling (PLS-SEM) was used to analyze the data in two phases: the measurement model assessment and structural model estimation. The measurement model was firstly evaluated via the PLS algorithm. Construct reliability was examined based on Cronbach’s α and composite reliability (CR), with the commonly accepted cutoff of 0.7 for both indicators (Adam et al., 2022; Hair et al., 2017; Ul Haq and Awan, 2020). As shown in Table 1, Cronbach’s α ranged from 0.936 to 0.959 and CR varied between 0.942 and 0.959 across three constructs (PQT, SCEn, IB), all exceeding the 0.7 threshold and satisfying internal reliability criteria.

**Table 6:** Results of HTMT criterion.

Variable	Perceived quality of teaching (PQT)	Student course engagement (SCEn)	Teacher interpersonal behaviour (IB)
Perceived Quality of Teaching (PQT)			
Student Course Engagement (SCEn)	0.843 CI.95 (0.827, 0.849)		
Teacher Interpersonal Behaviour (IB)	0.839 CI.95 (0.826, 0.845)	0.811 CI.95 (0.795, 0.829)	

Convergent validity was verified through standardized factor loadings (FL) and average variance extracted (AVE). Most items obtained factor loadings above the 0.7 benchmark, while a subset of items presented loadings ranging from 0.403 to 0.686, falling below the preferred cutoff. According to Fornell and Larcker (1981), items with loadings between 0.4 and 0.7 can be retained when the corresponding construct’s AVE is larger than 0.5. All three constructs yielded AVE values above 0.50 even with the full set of original measurement items retained (Hair et al., 2021). Collectively, these findings confirm satisfactory convergent validity for the measurement model (see Table 1).

Convergent validity was verified through standardized outer loadings (FL) and average variance extracted (AVE). According to Fornell and Larcker (1981) and Hair et al. (2021), items with outer loadings below 0.4 were iteratively removed. Items with loadings between 0.4 and 0.7 were retained only when the corresponding construct’s AVE exceeded 0.5. Most retained items obtained factor loadings above the preferred 0.7 benchmark; several items fell in the 0.4–0.7 range but were kept for theoretical reasons given satisfactory AVE values, as they capture unique conceptual dimensions of the target constructs and removing them would reduce scale comprehensiveness. Convergent-validity statistics for the final measurement model are presented in Table 1.

Discriminant validity was further assessed using the heterotrait-monotrait ratio (HTMT) criterion with 95% BCa bootstrap confidence intervals (Henseler et al., 2015). All HTMT point estimates are below the conventional 0.85 threshold. While the confidence-interval upper bound for PQT-IB approaches the 0.85 cutoff, all upper bounds remain below this threshold. Collectively, the results support acceptable discriminant validity; we nevertheless interpret structural-model outcomes with appropriate caution given the near-threshold value (Table 6).

#### Direct relationships in the structural model

In the third phase of data analysis, a structural model or path analysis was conducted using Smart PLS 4.0 and validated via bootstrapping. This phase immediately followed the assessment of the measurement model. The significance of the relationships was illustrated using path analysis, which delineates both direct and indirect effects; notably, direct effects correspond to ordinary linear regression. The path diagram was generated through 5,000 bootstrapping iterations to verify statistical significance.

We estimated three nested models to evaluate incremental explanatory power. Model 1 included only PQT as predictor (*R*^2^ = 0.644, adjusted-*R*^2^ = 0.643). Model 2 added the main effect of IB (*R*^2^ = 0.700, adjusted-*R*^2^ = 0.698). The full moderated Model 3 incorporated PQT, IB, and the latent interaction term PQT × IB, yielding *R*^2^ = 0.708, adjusted-*R*^2^ = 0.706. Adding IB increased explained variance by Δ*R*^2^ = 0.056 (adjusted-Δ*R*^2^ = 0.055); further adding the interaction term produced a small additional increment of Δ*R*^2^ = 0.008 (adjusted-Δ*R*^2^ = 0.008). Therefore, *R*^2^ = 0.708 reflects the collective explanatory power of the complete set of predictors rather than PQT alone.

The latent interaction PQT × IB was statistically significant [*β* = 0.068, *SE =* 0.033, *t =* 2.073, *p =* 0.038, 95% BCa CI (0.004, 0.136)]. Nevertheless, the coefficient magnitude and its *f* ^2^ = 0.030 indicate a small substantive effect. Simple slope numerical results are fully reported in Table 7.

**Table 7:** Simple slope analysis for the moderating effect of IB.

Level of IB	Slope (PQT → SCEn)
IB (−1 SD, low)	0.335
IB (Mean, average)	0.403
IB (+1 SD, high)	0.471

**Figure 1:**
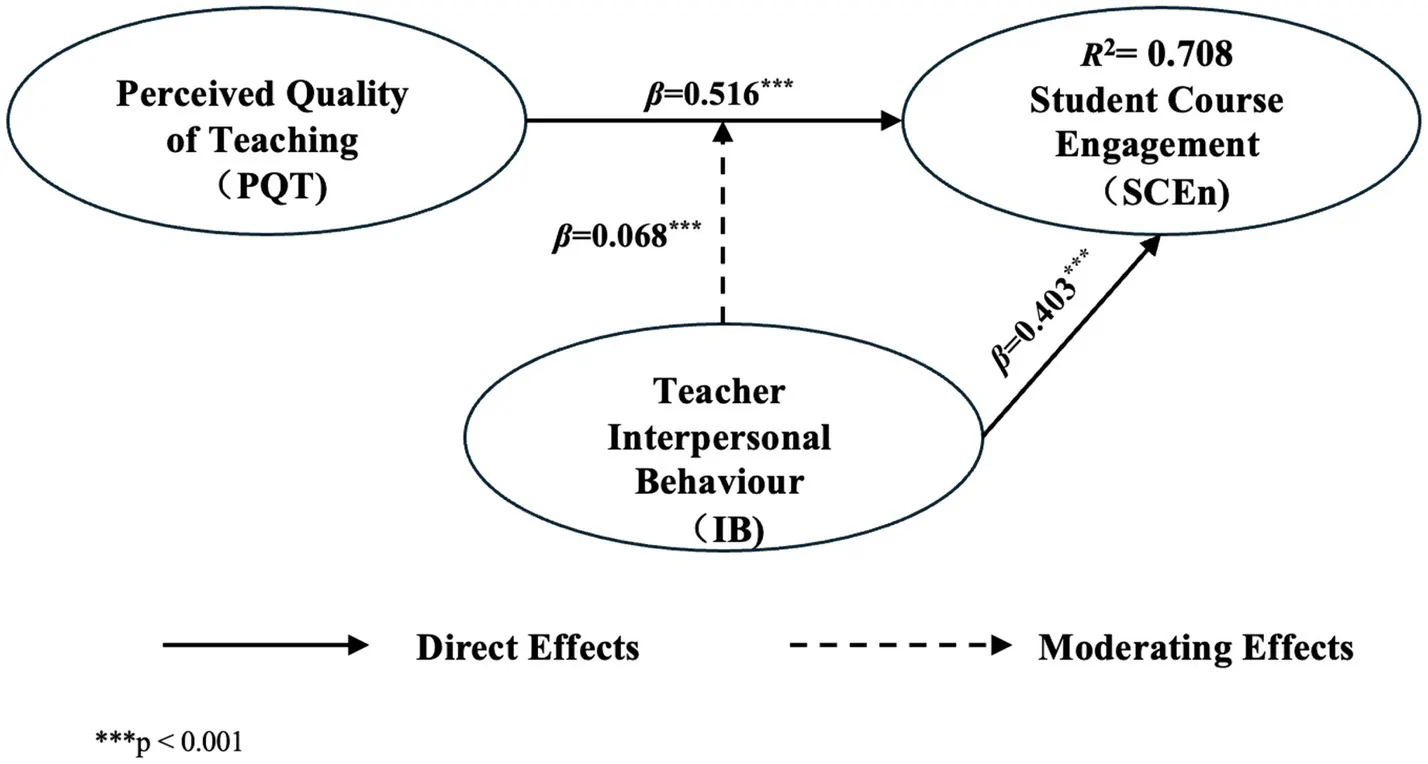
Estimated structural model results.

Analysis of the model revealed a significant positive relationship between PQT and SCEn (*β* = 0.516, *t* = 8.851), thereby supporting Hypothesis H1. This outcome demonstrates the robust impact of PQT in enhancing SCEn, as detailed in Table 8 and Figures 1, 2.

**Table 8:** Resulting direct relationships.

Structural paths	Path. Coeff.	*SE*	*t*-Value	*p*-values	*f* ^2^
PQT → SCEn	0.516	0.058	8.851	<0.001	0.326
IB → SCEn	0.403	0.059	6.858	<0.001	0.201
PQT × IB → SCEn	0.068	0.033	2.073	0.038	0.030

*R*^2^ = 0.708 (adjusted *R*^2^ = 0.706).

**Figure 2:**
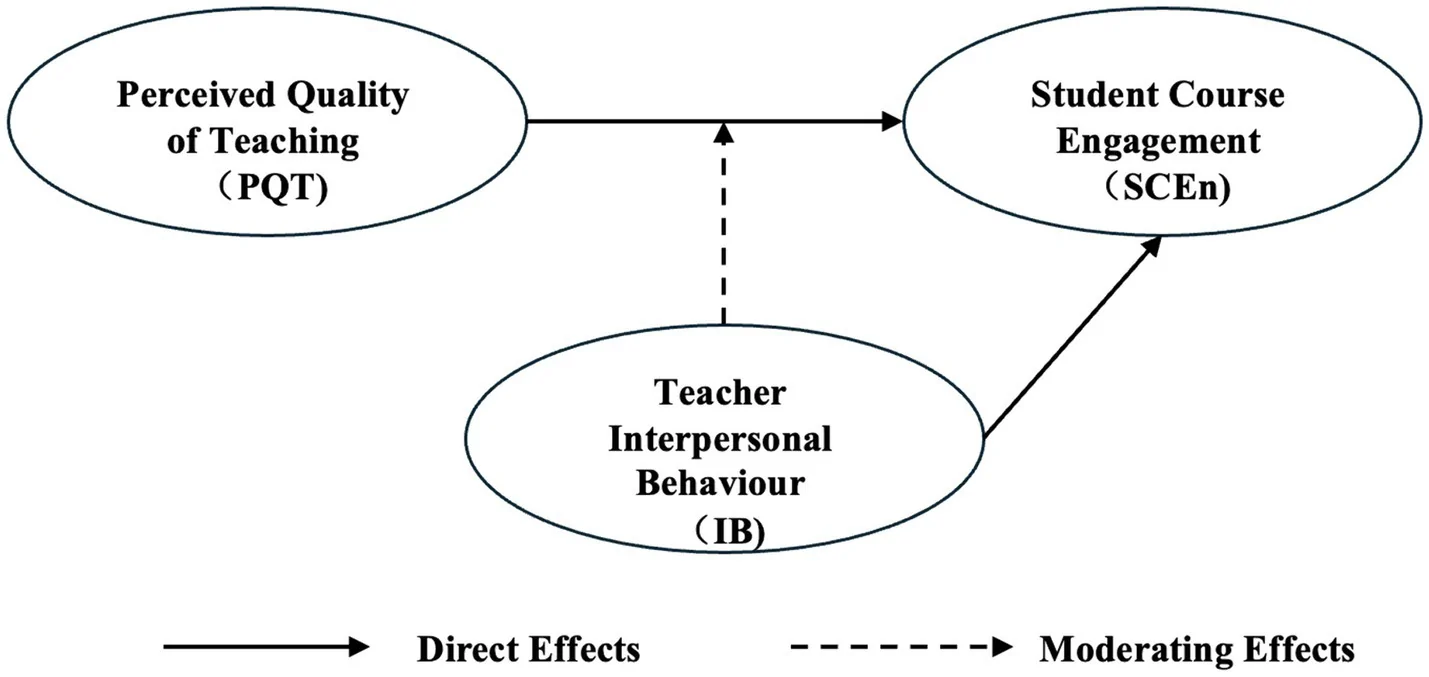
Conceptual research framework.

#### Moderating effects

The subsequent objective of the study was to investigate the moderating role of Teacher Interpersonal Behaviour on the relationship between Perceived Quality of Teaching and Student Course Engagement. To evaluate the interaction effects of the moderator, PLS-SEM was employed, and the orthogonalization method was applied following Henseler and Chin’s (2010) recommendations to identify the moderating influence of Teacher Interpersonal Behaviour. The significance of this moderator was assessed via bootstrapping with 5,000 resamples. Based on these results, the interaction term was computed by multiplying the moderator by the exogenous variable (Adam and Mahadi, 2018). As shown in Table 8, Teacher Interpersonal Behaviour significantly moderated the relationship between Perceived Quality of Teaching and Student Course Engagement. As Teacher Interpersonal Behaviour increases, so does the influence of Perceived Quality of Teaching on Student Course Engagement. Therefore, Hypothesis H2 was supported.

## Discussion

This study examined the relationship between perceived quality of teaching (PQT) and student course engagement (SCEn), with a particular focus on the moderating role of teacher interpersonal Behaviour (IB) in the context of entrepreneurship education. Overall, the findings indicate that students perceived quality of teaching, interpersonal Behaviour, and their own engagement favorably, suggesting a generally supportive instructional environment. These empirical outcomes are interpreted below against the literature-based research gaps and the integrated theoretical perspective (SDT, SET, and Incremental Theory of Learning) laid out in the Literature Review and Theoretical Framework section.

More importantly, the results demonstrate that PQT is a strong predictor of SCEn, reinforcing the central role of instructional quality in fostering students’ behavioral, emotional, and cognitive engagement. This finding aligns with prior research highlighting that well-structured content, clear goals, appropriate assessment, and meaningful learning activities are fundamental drivers of engagement in higher education (Banks and Smyth, 2021; Wang, 2024). From a theoretical perspective, this result is consistent with Dweck’s (1999) Incremental Theory of Learning, which posits that learning engagement can be continuously enhanced through supportive and effective instructional practices (see Figure 3).

**Figure 3:**
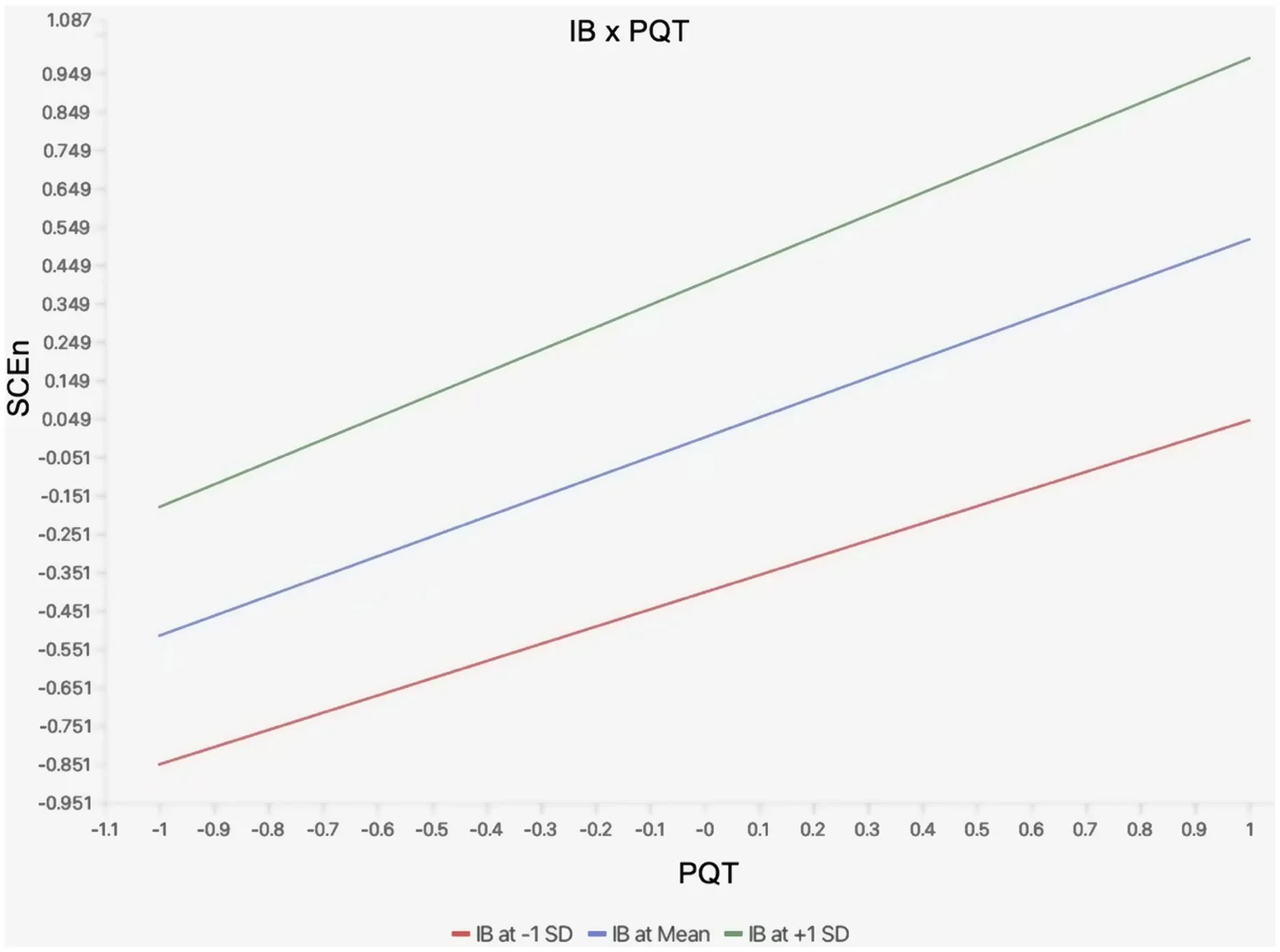
Interaction plot: the effect of PQT on SCEn at low versus high levels of IB.

Beyond this direct relationship, this study identifies a statistically significant moderating role of teacher interpersonal behaviour. It should be noted, however, that the interaction yields only a small substantive effect (*f* ^2^ = 0.030, Δ*R*^2^ = 0.008). Although the pattern aligns with our theoretical expectation, its practical incremental contribution is modest. This finding indicates that supportive teacher interpersonal behaviour can slightly amplify the positive association between perceived quality of teaching and student course engagement, yet the main effects of PQT and IB are substantially more influential for shaping course engagement outcomes. This interaction effect extends prior work grounded in Self-Determination Theory (Ryan and Deci, 2000) and Self-Efficacy Theory (Bandura, 1997), both of which emphasize the importance of interpersonal support in satisfying students’ psychological needs and strengthening their motivation to engage. Consistent with Klem and Connell (2004) and more recent evidence (Ma et al., 2024), the results highlight that instructional quality alone may be insufficient if it is not embedded within positive interpersonal classroom climates.

Taken together, these findings move beyond a purely instructional or relational explanation of engagement and demonstrate that teaching quality and interpersonal behaviour function synergistically. This integrative perspective is particularly relevant for entrepreneurship education, where experiential, collaborative, and self-directed learning intensifies students’ dependence on both pedagogical structure and interpersonal support (Mei et al., 2020).

### Theoretical implications

Building on the theoretical reasoning elaborated in the Literature Review and Theoretical Framework, our findings further advance existing scholarship in several important ways. This study contributes to the literature on student engagement and instructional effectiveness in several important ways. First, it empirically confirms perceived quality of teaching as a central determinant of student course engagement within entrepreneurship education, thereby extending engagement research into a domain characterized by experiential and practice-oriented learning. While previous studies have acknowledged the importance of teaching quality, this research demonstrates its explanatory power within a non-traditional instructional context that emphasizes autonomy and active engagement.

By introducing teacher interpersonal behaviour as a moderator, the study advances existing theoretical frameworks by highlighting the conditional nature of teaching quality effects.

It should be noted that our dataset cannot empirically test the intra-psychological motivational mechanisms postulated by SDT and SET, given that these internal psychological processes were not directly measured in the present survey and serve merely as interpretive background for the observed associations. The integration of Incremental Theory of Learning (Dweck, 1999) with instructional and interpersonal dimensions provides a more holistic explanation of engagement development. The results suggest that students’ engagement is not only shaped by instructional design but is also dynamically influenced by how teaching quality is enacted through daily interpersonal exchanges. By empirically modeling this interaction, the study offers a refined theoretical understanding of how cognitive, motivational, and social mechanisms jointly operate in entrepreneurship education.

Building on the above theoretical insights, the present study also highlights why such mechanisms matter specifically for entrepreneurship-education contexts. Lastly, entrepreneurship-education learning is inherently characterized by uncertainty, experiential experimentation, student agency, collaborative problem-solving, and idea pitching (Mei et al., 2020). These contextual features heighten students’ psychological needs for autonomy-supportive teacher behaviours. Accordingly, the interplay between teaching quality and teacher interpersonal behaviour carries unique significance within entrepreneurship education, rather than constituting a simple contextual replication of generic educational-psychology findings.

### Practical implications

The findings yield several actionable implications for higher education institutions and policymakers, particularly those responsible for entrepreneurship education. It should be emphasised that the present study only measures student course engagement and student perceptions of teaching and teacher interpersonal behaviour; we do not directly assess entrepreneurial-related outcomes such as entrepreneurial competence, entrepreneurial intention, opportunity recognition, venture-creation, employability, or entrepreneurial self-efficacy. Any links to entrepreneurial competencies should therefore be regarded as plausible potential future outcomes rather than demonstrated consequences from this dataset.

First, the strong positive association between perceived quality of teaching and student course engagement underscores the need for systematic investment in instructional design within entrepreneurship-education courses. Universities should support teaching practices that emphasize clarity of goals, authentic assessment, and experiential learning activities suited for entrepreneurship-focused coursework.

For the Chinese higher-education context, this is particularly salient given that entrepreneurship education is a mandatory undergraduate requirement delivered across all disciplines. Many compulsory EE classes in China are taught by instructors without dedicated entrepreneurship-related training or practical entrepreneurial experience. Therefore, local universities cannot rely merely on policy mandates; they need to refine course content and experiential activities tailored for large mixed-discipline cohorts of first-year undergraduates, rather than adopting elite-oriented entrepreneurship curricula designed for business majors only.

The moderating role of teacher interpersonal behaviour suggests that professional development initiatives should move beyond technical pedagogy and explicitly address interpersonal competencies. Entrepreneurship educators may need to combine sound instructional design with supportive interpersonal practices, particularly during uncertain project work, entrepreneurial idea development, group activities, pitching, feedback, and other autonomy-demanding learning tasks. Training programs can incorporate modules on autonomy-supportive communication, constructive feedback, and relational sensitivity to foster higher levels of student course engagement.

This recommendation carries special local relevance for Chinese university classrooms shaped by traditional hierarchical teacher-student norms. Local faculty development workshops should help Chinese EE instructors balance traditional authoritative teaching habits with autonomy-supportive interpersonal strategies, especially when teaching large compulsory classes with heterogeneous first-year students from diverse disciplinary backgrounds.

At the institutional level, these findings support policies that encourage reflective teaching evaluation systems, combining student feedback on both instructional quality and interpersonal behaviour. Such evidence-based approaches can help institutions create learning environments that foster intrinsic motivation and sustained student course engagement. Improvements in entrepreneurial-related competencies represent a plausible downstream possibility that requires further dedicated empirical investigation.

For Chinese universities, existing teaching evaluation systems mostly focus on general teaching indicators; institutional evaluation mechanisms could be adjusted to also collect student feedback specifically targeting entrepreneurship courses, covering both pedagogical quality and teacher-student interpersonal interactions. This provides local university administrators with context-specific evidence to improve the implementation of nationwide mandatory entrepreneurship-education requirements.

## Limitations and future research recommendations

Despite its contributions, this study has several limitations. First, the sample was limited to first-year students from 16 schools in a comprehensive university in China, which may restrict the generalizability of the findings. Future research should include students from different academic years, institutions, and cultural contexts to enhance external validity. Secondly, the cross-sectional design limits causal interpretation. Longitudinal or experimental studies are recommended to examine how perceived quality of teaching, interpersonal behaviour, and engagement evolve over time and to identify potential long-term effects. Thirdly, the reliance on self-reported data may introduce response bias. Future studies could incorporate multi-source data, such as classroom observations or instructor assessments, to strengthen measurement validity. Fourthly, formal measurement invariance testing was not performed; autonomy-support related constructs may carry culture-specific meanings in Chinese higher-education. Finally, while this study focused on teacher interpersonal behaviour as a moderator, future research could examine additional mediators or moderators, such as intrinsic motivation, learning strategies, or institutional support, and extend the model to other academic disciplines.

Additionally, the Interpersonal Behaviour Questionnaire combines need-supportive and need-thwarting sub-dimensions into a single aggregated latent score in the present analysis. This aggregation limits fine-grained interpretation of whether supportive versus thwarting teacher behaviours exert differential moderating effects. Future research is encouraged to separate need-supportive and need-thwarting interpersonal behaviours and test their respective roles. In addition, several indicators for the adapted PQT and IB scales fall at marginal loading thresholds; future research may further refine item wording to improve indicator performance.

Nevertheless, shared-source bias may have inflated observed associations and may partly contribute to the comparatively high *R*^2^ value observed in the model. Future investigations should adopt temporally separated survey waves, multi-source data such as instructor ratings or objective behavioural classroom indicators, and classroom observation data to mitigate common-method bias and better evaluate potential causal ordering, including reverse-direction possibilities.

Another potential limitation concerns nested data structure: students are nested within classes and instructors, with multiple students rating the same teacher. Treating all 456 observations as fully independent may underestimate standard errors, a notable concern because teacher interpersonal behaviour is our moderator. Our dataset lacks complete respondent-to-instructor-class identifiers. Without these grouping keys, we cannot compute intra-class correlation coefficients (ICC), multilevel-SEM, or cluster-robust standard-error adjustments. Thus, the PLS-SEM analysis does not account for potential clustering. Readers should interpret path coefficients and significance with caution. Future studies should collect instructor-class identifiers, calculate ICC, and adopt multilevel modelling or cluster-robust standard errors if clustering exists.

## Conclusion

This study examined the relationship between perceived quality of teaching and student course engagement, highlighting the moderating role of teacher interpersonal behaviour in entrepreneurship education. The findings show that higher perceived quality of teaching was associated with higher reported student course engagement, and that this association varied modestly according to perceived teacher interpersonal behaviour. These cross-sectional associations point to the interplay between instructional quality and interpersonal classroom dynamics among first-year entrepreneurship-education students. By integrating instructional quality with interpersonal dynamics, the study advances theoretical understanding of engagement and offers practical guidance for improving teaching practices. These results underscore the importance of combining effective pedagogy with positive teacher–student relationships to foster engaging and meaningful learning experiences in higher education.

## Data Availability

The raw data supporting the conclusions of this article will be made available by the authors, without undue reservation.
