# Supplementary material for: Perceived quality of teaching and course engagement in entrepreneurship education: the moderating role of teacher interpersonal behaviour among first-year undergraduates at a Chinese university
Source: Front Psychol. 2026 Oct 19;17:1936338. doi: 10.3389/fpsyg.2026.1936338 (PMC13724435; doi:10.3389/fpsyg.2026.1936338)
Supplement: Supplementary file 1 [file Supplementary_file_1.DOCX]

**Supplementary Material: Instrument adaptation documentation**

This supplementary file provides full documentation for the three survey instruments used in this study. It includes retained item wording (Chinese and back‑translated English), modification log for the adapted PQTQ scale, marking of reverse‑scored items for IBQ, full CVR results, and full text of deleted items.

PQTQ was substantially adapted from the Student Course Experience Questionnaire (SCExQ, Ginns et al., 2007) for use in a compulsory entrepreneurship‑course setting; it is not a simple renaming of the original scale.

SCEnQ (Handelsman et al., 2005) and IBQ (Rocchi et al., 2017) were translated and subjected to content‑validity screening via CVR; no major contextual rewriting of item stems was performed.

Response scale for all questionnaires: 1 = Strongly Disagree; 2 = Disagree; 3 = Partly Agree; 4 = Agree; 5 = Strongly Agree

**Table S1 Retained items of PQTQ (adapted from SCExQ; Ginns et al., 2007)**

| PQTQ Item ID | Chinese administered item | Back‑translated English | Original SCExQ Item ID |
| --- | --- | --- | --- |
| PQTQ1 | 老师通常会对我的学习情况给予有益的反馈 | The teaching staff normally give me helpful feedback on how I am doing | SCExQ‑3 |
| PQTQ2 | 这门创业必修课的老师激励着让我做到最好 | The teaching staff of this compulsory entrepreneurship course motivate me to do my best work | SCExQ‑8 |
| PQTQ3 | 老师很能够理解我在课程中可能遇到的困难 | The staff make a real effort to understand difficulties I may be having with my work | SCExQ‑16 |
| PQTQ4 | 我的老师在讲解知识和技能方面非常擅长 | My lecturers are extremely good at explaining things | SCExQ‑19 |
| PQTQ6 | 老师花了很多时间对我的课程表现进行评论 | The staff put a lot of time into commenting on my work | SCExQ‑27 |
| PQTQ7 | 我清楚地知道在这门必修创业课程中我要做些什么，以及老师对我的期望是什么 | I have usually had a clear idea of where I am going and what is expected of me in compulsory entrepreneurship course | SCExQ‑6 |
| PQTQ8 | 这门课的课程标准很清晰易懂 | It is always easy to know the standard of work expected | SCExQ‑12 |
| PQTQ9 | 老师从一开始就跟我们明确了他对我们的期望 | The staff made it clear right from the start what they expected from students | SCExQ‑25 |
| PQTQ15 | 这门课的作业量很大* | The workload is too heavy * | SCExQ‑4 |
| PQTQ16 | 我通常有足够的时间来理解我必须学习的东西 | I am generally given enough time to understand the things I have to learn | SCExQ‑15 |
| PQTQ18 | 这门必修创业课程培养了我团队合作的能力 | The compulsory entrepreneurship course has helped me develop my ability to work as a team member. | SCExQ‑5 |
| PQTQ19 | 这门必修创业课程提高了我的分析能力 | This compulsory entrepreneurship course has sharpened my analytic skills | SCExQ‑9 |
| PQTQ20 | 通过学习这门必修创业课程，使我对解决不熟悉的问题有信心 | As a result of this compulsory entrepreneurship course  , I feel confident about tackling unfamiliar problems | SCExQ‑10 |
| PQTQ21 | 这门必修创业课程培养了我问题解决的能力 | This compulsory entrepreneurship course has developed my problem-solving skills | SCExQ‑18 |
| PQTQ22 | 这门必修创业课程提高了我的书面表达的通能力 | The compulsory entrepreneurship course has improved my skills in written communication | SCExQ‑22 |
| PQTQ23 | 这门必修创业课程培养了我规划自己的学习或工作的能力 | My compulsory entrepreneurship course has helped me to develop the ability to plan my own work | SCExQ‑23 |

*Note:* * = reversed item. This adapted version targets the compulsory entrepreneurship course, rather than the whole degree programme.

**Table S2 Item‑by‑item modification log for SCExQ‑to‑PQTQ**

| Original SCExQ ID | Original item wording (English) | Modification made in adapted PQTQ | Rationale for modification |
| --- | --- | --- | --- |
| SCExQ‑1 | The teaching staff normally give me helpful feedback on how I am doing | No modification; item retained with context updated to the entrepreneurship course | The original item does not mention “degree‑course‑wide” content |
| SCExQ‑2 | The teaching staff of this degree course motivate me to do my best work | Replace “this degree course” with “in this compulsory entrepreneurship course” | Original item refers to whole‑degree programme; revised to target the focal single entrepreneurship course |
| SCExQ‑3 | The staff make a real effort to understand difficulties I may be having with my work | No modification; item retained with context updated to the entrepreneurship course | The original item does not mention “degree‑course‑wide” content |
| SCExQ‑4 | My lecturers are extremely good at explaining things | No modification; item retained with context updated to the entrepreneurship course | The original item does not mention “degree‑course‑wide” content |
| SCExQ‑5 | The teaching staff work hard to make their subjects interesting | Item deleted | Eliminated due to negative CVR value in content‑validity assessment |
| SCExQ‑6 | The staff put in a lot of time into commenting on my work | No modification; item retained with context updated to the entrepreneurship course | The original item does not mention “degree‑course‑wide” content |
| SCExQ‑7 | I have usually had a clear idea of where I am going and what is expected of me in this degree course | Replace “in this degree course” with “in this compulsory entrepreneurship course” | Original item refers to whole‑degree programme; revised to target the focal single entrepreneurship course |
| SCExQ‑8 | It is always easy to know the standard of work expected | No modification; item retained with context updated to the entrepreneurship course | The original item does not mention “degree‑course‑wide” content |
| SCExQ‑9 | The staff made it clear right from the start what they expected from students | No modification; item retained with context updated to the entrepreneurship course | The original item does not mention “degree‑course‑wide” content |
| SCExQ‑10 | It has often been hard to discover what is expected of me in this degree course | Item deleted；& Replace “in this degree course” with “ in this compulsory entrepreneurship course” | Eliminated due to negative CVR value in content‑validity assessment |
| SCExQ‑11 | The staff seem more interested in testing what I have memorized than what I have understood * | Item deleted；pre‑deletion loading = 0.394 | Eliminated due to outer loading < 0.4 threshold |
| SCExQ‑12 | Too many staff ask me questions just about facts * | Item deleted；pre‑deletion loading = 0.285 | Eliminated due to outer loading < 0.4 threshold |
| SCExQ‑13 | To do well in this degree all you really need is a good memory * | Item deleted | Eliminated due to negative CVR value in content‑validity assessment |
| SCExQ‑14 | There is a lot of pressure on me as a student in this degree course * | Item deleted；pre‑deletion loading = 0.399 | Eliminated due to outer loading < 0.4 threshold |
| SCExQ‑15 | The workload is too heavy * | No modification; item retained with context updated to the entrepreneurship course | The original item does not mention “degree‑course‑wide” content |
| SCExQ‑16 | I am generally given enough time to understand the things I have to learn | No modification; item retained with context updated to the entrepreneurship course | The original item does not mention “degree‑course‑wide” content |
| SCExQ‑17 | The sheer volume of work to be got through in this degree  means it can’t all be thoroughly comprehended * | Item deleted | Eliminated due to negative CVR value in content‑validity assessment |
| SCExQ‑18 | The degree course has helped me develop my ability to work as a team member. | Replace “The degree course” with “This compulsory entrepreneurship course” | Original item refers to whole‑degree programme; revised to target the focal single entrepreneurship course |
| SCExQ‑19 | The degree course has sharpened my analytic skills | Replace “The degree course” with “This compulsory entrepreneurship course” | Original item refers to whole‑degree programme; revised to target the focal single entrepreneurship course |
| SCExQ‑20 | As a result of my degree course, I feel confident about tackling unfamiliar problems | Replace “my degree course” with “this compulsory entrepreneurship course” | Original item refers to whole‑degree programme; revised to target the focal single entrepreneurship course |
| SCExQ‑21 | The degree course has developed my problem-solving skills | Replace “The degree course” with “This compulsory entrepreneurship course” | Original item refers to whole‑degree programme; revised to target the focal single entrepreneurship course |
| SCExQ‑22 | The degree course has improved my skills in written communication | Replace “The degree course” with “This compulsory entrepreneurship course” | Original item refers to whole‑degree programme; revised to target the focal single entrepreneurship course |
| SCExQ‑23 | My degree course has helped me to develop the ability to plan my own work | Replace “My degree course” with “This compulsory entrepreneurship course” | Original item refers to whole‑degree programme; revised to target the focal single entrepreneurship course |

*Note:* * = reversed item.

**Table S3 Full item‑level screening documentation: SCEnQ (Handelsman et al., 2005)**

| SCEnQ Item ID | Chinese administered item（正式中文施测题项） | Back‑translated English（回译英文） | Original SCEnQ Item ID | Modification made in adapted SCEnQ | Rationale for modification |
| --- | --- | --- | --- | --- | --- |
| SCEnQ1 | 我确保定期学习 | Making sure to study on a regular basis | SCEnQ‑1 | No modification; item retained with context updated to the entrepreneurship course | The original item does not mention “degree‑course‑wide” content |
| SCEnQ2 | 我努力地去学好这门课 | Putting forth effort | SCEnQ‑2 | No modification; item retained with context updated to the entrepreneurship course | The original item does not mention “degree‑course‑wide” content |
| SCEnQ3 | 我会去完成所有作业题目 | Doing all the homework problems | SCEnQ‑3 | No modification; item retained with context updated to the entrepreneurship course | The original item does not mention “degree‑course‑wide” content |
| SCEnQ4 | 我能够坚持去阅读 | Staying up on the readings | SCEnQ‑4 | No modification; item retained with context updated to the entrepreneurship course | The original item does not mention “degree‑course‑wide” content |
| SCEnQ5 | 我能够在课间复习课堂学习笔记，确保我理解所学习的内容 | Looking over class notes between classes to make sure I understand the material | SCEnQ‑5 | Item deleted | Eliminated due to negative CVR value in content‑validity assessment |
| SCEnQ6 | 有条理地去学习这门课 | Being organized | SCEnQ‑6 | Item deleted | Eliminated due to negative CVR value in content‑validity assessment |
| SCEnQ7 | 我会在课堂上做好笔记 | Taking good notes in class | SCEnQ‑7 | Item deleted | Eliminated due to negative CVR value in content‑validity assessment |
| SCEnQ8 | 课堂上我会认真听讲 | Listening carefully in class | SCEnQ‑8 | Item deleted | Eliminated due to negative CVR value in content‑validity assessment |
| SCEnQ9 | 我出席了这门课程的每一次课 | Coming to class every day | SCEnQ‑9 | No modification; item retained with context updated to the entrepreneurship course | The original item does not mention “degree‑course‑wide” content |
| SCEnQ10 | 我会找方法让课程内容与我的生活实践相关联 | Finding ways to make the course material relevant to my life | SCEnQ‑10 | No modification; item retained with context updated to the entrepreneurship course | The original item does not mention “degree‑course‑wide” content |
| SCEnQ11 | 我会将课程内容应用到我的生活实践中 | Applying course material to my life | SCEnQ‑11 | No modification; item retained with context updated to the entrepreneurship course | The original item does not mention “degree‑course‑wide” content |
| SCEnQ12 | 我会想办法让自己对这门课感兴趣 | Finding ways to make the course interesting to me | SCEnQ‑12 | No modification; item retained with context updated to the entrepreneurship course | The original item does not mention “degree‑course‑wide” content |
| SCEnQ13 | 我会在课间思考复习这门课程 | Thinking about the course between meetings | SCEnQ‑13 | Item deleted | Eliminated due to negative CVR value in content‑validity assessment |
| SCEnQ14 | 我渴望学好这门课 | Really desiring to learn the material | SCEnQ‑14 | No modification; item retained with context updated to the entrepreneurship course | The original item does not mention “degree‑course‑wide” content |
| SCEnQ15 | 我会在课堂上举手发言 | Raising my hand in class | SCEnQ‑15 | No modification; item retained with context updated to the entrepreneurship course | The original item does not mention “degree‑course‑wide” content |
| SCEnQ16 | 当我不理解时，我会问老师问题 | Asking questions when I don’t understand the instructor | SCEnQ‑16 | Item deleted | Eliminated due to negative CVR value in content‑validity assessment |
| SCEnQ17 | 在课程中我能感到快乐 | Having fun in class | SCEnQ‑17 | No modification; item retained with context updated to the entrepreneurship course | The original item does not mention “degree‑course‑wide” content |
| SCEnQ18 | 我会积极参与小组讨论 | Participating actively in small‑group Discussions | SCEnQ‑18 | Item deleted | Eliminated due to negative CVR value in content‑validity assessment |
| SCEnQ19 | 我会向老师请教创业相关问题xml | Going to the professor’s office during office hours to review assignments or tests or to ask questions | SCEnQ‑19 | No modification; item retained with context updated to the entrepreneurship course | The original item does not mention “degree‑course‑wide” content |
| SCEnQ20 | 我会帮助其他同学 | Helping fellow students | SCEnQ‑20 | No modification; item retained with context updated to the entrepreneurship course | The original item does not mention “degree‑course‑wide” content |
| SCEnQ21 | 这门课取得了好成绩 | Getting a good grade | SCEnQ‑21 | No modification; item retained with context updated to the entrepreneurship course | The original item does not mention “degree‑course‑wide” content |
| SCEnQ22 | 我考试考的很好 | Doing well on the tests（ | SCEnQ‑22 | Item deleted | Eliminated due to negative CVR value in content‑validity assessment |
| SCEnQ23 | 我相信自己能学好并在课堂上表现出色 | Being confident that I can learn and do well in the class | SCEnQ‑23 | No modification; item retained with context updated to the entrepreneurship course | The original item does not mention “degree‑course‑wide” content |

*Not**e:* Only item deletion was conducted; no contextual rewriting of item stems.

**Table S4 Full item‑level screening documentation: IBQ (Rocchi et al., 2017)**

| IBQ Item ID | Chinese administered item | Back‑translated English | Original SCEnQ Item ID | Modification made in adapted IBQ | Rationale for modification |
| --- | --- | --- | --- | --- | --- |
| IBQ1 | 给我自主选择的自由 | Give me the freedom to make my own choices | IBQ1 | No modification; item retained with context updated to the entrepreneurship course | The original item does not mention “degree‑course‑wide” content |
| IBQ2 | 支持我的决定 | Support my decisions | IBQ2 | No modification; item retained with context updated to the entrepreneurship course | The original item does not mention “degree‑course‑wide” content |
| IBQ3 | 支持我为我自己做出的选择 | Support the choices that I make for myself | IBQ3 | No modification; item retained with context updated to the entrepreneurship course | The original item does not mention “degree‑course‑wide” content |
| IBQ4 | 鼓励我自己做决定 | Encourage me to make my own decisions | IBQ4 | No modification; item retained with context updated to the entrepreneurship course | The original item does not mention “degree‑course‑wide” content |
| IBQ5 | 强迫我按他们的方式做事* | Pressure me to do things their way* | IBQ5 | Item deleted；pre‑deletion loading = 0.398 | Eliminated due to outer loading < 0.4 threshold |
| IBQ6 | 把他们的意见强加于我* | Impose their opinions on me* | IBQ6 | Item deleted；pre‑deletion loading = 0.292 | Eliminated due to outer loading < 0.4 threshold |
| IBQ7 | 迫使我采纳某些行为方式* | Pressure me to adopt certain behaviours* | IBQ7 | No modification; item retained with context updated to the entrepreneurship course | The original item does not mention “degree‑course‑wide” content |
| IBQ8 | 限制我的选择* | Limit my choices* | IBQ8 | No modification; item retained with context updated to the entrepreneurship course | The original item does not mention “degree‑course‑wide” content |
| IBQ9 | 鼓励我提升我的技能 | Encourage me to improve my skills | IBQ9 | No modification; item retained with context updated to the entrepreneurship course | The original item does not mention “degree‑course‑wide” content |
| IBQ10 | 提供有价值的反馈 | Provide valuable feedback | IBQ10 | No modification; item retained with context updated to the entrepreneurship course | The original item does not mention “degree‑course‑wide” content |
| IBQ11 | 肯定我有能力达成我的目标 | Acknowledge my ability to achieve my goals | IBQ11 | No modification; item retained with context updated to the entrepreneurship course | The original item does not mention “degree‑course‑wide” content |
| IBQ12 | 告诉我我可以做成一些事情 | Tell me that I can accomplish things | IBQ12 | No modification; item retained with context updated to the entrepreneurship course | The original item does not mention “degree‑course‑wide” content |
| IBQ13 | 指出我很有可能会失败* | Point out that I will likely fail* | IBQ13 | Item deleted；pre‑deletion loading = 0.017 | Eliminated due to outer loading < 0.4 threshold |
| IBQ14 | 向我传递我是无能的这种信息* | Send me the message that I am incompetent* | IBQ14 | Item deleted；pre‑deletion loading =- 0.060 | Eliminated due to outer loading < 0.4 threshold |
| IBQ15 | 怀疑我提升的能力* | Doubt my capacity to improve* | IBQ15 | Item deleted；pre‑deletion loading = 0.068 | Eliminated due to outer loading < 0.4 threshold |
| IBQ16 | 质疑我克服挑战的能力* | Question my ability to overcome challenges* | IBQ16 | Item deleted；pre‑deletion loading =0.059 | Eliminated due to outer loading < 0.4 threshold |
| IBQ17 | 对我所做的事情感兴趣 | Are interested in what I do | IBQ17 | No modification; item retained with context updated to the entrepreneurship course | The original item does not mention “degree‑course‑wide” content |
| IBQ18 | 花时间去了解我 | Take the time to get to know me | IBQ18 | No modification; item retained with context updated to the entrepreneurship course | The original item does not mention “degree‑course‑wide” content |
| IBQ19 | 真诚地享受和我在一起的时光 | Honestly enjoy spending time with me | IBQ19 | No modification; item retained with context updated to the entrepreneurship course | The original item does not mention “degree‑course‑wide” content |
| IBQ20 | 与我产生亲近感、有共鸣 | Relate to me | IBQ20 | No modification; item retained with context updated to the entrepreneurship course | The original item does not mention “degree‑course‑wide” content |
| IBQ21 | 当我情绪低落时不安慰我* | Do not comfort me when I am feeling low* | IBQ21 | No modification; item retained with context updated to the entrepreneurship course | The original item does not mention “degree‑course‑wide” content |
| IBQ22 | 和我在一起时显得疏远* | Are distant when we spend time together* | IBQ22 | Item deleted | Eliminated due to negative CVR value in content‑validity assessment |
| IBQ23 | 不与我建立联系* | Do not connect with me* | IBQ23 | No modification; item retained with context updated to the entrepreneurship course | The original item does not mention “degree‑course‑wide” content |
| IBQ24 | 不关心我* | Do not care about me* | IBQ24 | No modification; item retained with context updated to the entrepreneurship course | The original item does not mention “degree‑course‑wide” content |

*Note:* * = reversed item; only item deletion was conducted; no contextual rewriting of item stems.

**Note on deleted items**

**Deleted items from PQTQ (7 items)**

1. SCExQ‑5: The teaching staff work hard to make their subjects interesting.（Chinese：老师很努力以使他的课程变得有趣）

2. SCExQ‑10: It has often been hard to discover what is expected of me in this degree course. （Chinese：我经常很难弄清楚这门课程对我的期望是什么）

3. SCExQ‑11: The staff seem more interested in testing what I have memorized than what I have understood. （Chinese：老师们似乎更感兴趣的是测试我记住了什么，而不是我理解了什么）

4. SCExQ‑12: Too many staff ask me questions just about facts. （Chinese：老师总是问我知识点理解的问题）

5. SCExQ‑13: To do well in this degree all you really need is a good memory. （Chinese：要在这个学位课程中表现出色，实际上你只需要有一个好的记忆力）

6. SCExQ‑14: There is a lot of pressure on me as a student in this degree course. （Chinese：要在这个学位课程中表现出色，实际上你只需要有一个好的记忆力）作为这个学位课程的学生，我承受着很大的压力

7. SCExQ‑17: The sheer volume of work to be got through in this degree means it can’t all be thoroughly comprehended. （Chinese：这个学位课程的工作量如此之大，以至于无法全部彻底理解）

**Deleted items from SCEnQ (8 items)**

1. SCEnQ‑5: Looking over class notes between classes to make sure I understand the material（Chinese：我能够在课间复习课堂学习笔记，确保我理解所学习的内容）

2. SCEnQ‑6: Being organized. （Chinese：有条理地去学习这门课）

3. SCEnQ‑7: Taking good notes in class. （Chinese：我会在课堂上做好笔记）

4. SCEnQ‑8: Listening carefully in class. （Chinese：课堂上我会认真听讲）

5. SCEnQ‑13: Thinking about the course between meetings. （Chinese：我会在课间思考复习这门课程）

6. SCEnQ‑16: Asking questions when I don’t understand the instructor. （Chinese：当我不理解时，我会问老师问题）

7. SCEnQ‑18: Participating actively in small‑group Discussions. （Chinese：我会积极参与小组讨论）

8. SCEnQ‑22: Doing well on the tests. （Chinese：我考试考的很好）

**Deleted items from IBQ (7 items)**

1. IBQ‑5: Pressure me to do things their way. （Chinese：强迫我按他们的方式做事）
2. IBQ‑6: Impose their opinions on me. （Chinese：把他们的意见强加于我）
3. IBQ‑13: Point out that I will likely fail. （Chinese：指出我很有可能会失败）
4. IBQ‑14: Send me the message that I am incompetent. （Chinese：向我传递我是无能的这种信息）
5. IBQ‑15: Doubt my capacity to improve. （Chinese：怀疑我提升的能力）
6. IBQ‑16: Question my ability to overcome challenges. （Chinese：质疑我克服挑战的能力）
7. IBQ‑22: Are distant when we spend time together（Chinese：和我在一起时显得疏远）
